# Dysregulation of estrogen receptor beta (ERβ), aromatase (CYP19A1), and ER co-activators in the middle frontal gyrus of autism spectrum disorder subjects

**DOI:** 10.1186/2040-2392-5-46

**Published:** 2014-09-09

**Authors:** Amanda Crider, Roshni Thakkar, Anthony O Ahmed, Anilkumar Pillai

**Affiliations:** Department of Psychiatry and Health Behavior, Medical College of Georgia, Georgia Regents University, 997 St. Sebastian Way, Augusta, GA 30912 USA; Department of Neuroscience and Regenerative Medicine, Medical College of Georgia, Georgia Regents University, 997 St. Sebastian Way, Augusta, GA 30912 USA

**Keywords:** Aromatase, Autism, Estrogen, Receptor, Sex

## Abstract

**Background:**

Autism spectrum disorders (ASD) are much more common in males than in females. Molecular alterations within the estrogen receptor (ER) signaling pathway may contribute to the sex difference in ASD, but the extent of such abnormalities in the brain is not known.

**Methods:**

Postmortem middle frontal gyrus tissues (13 ASD and 13 control subjects) were used. The protein levels were examined by western blotting. The gene expression was determined by qRT-PCR.

**Results:**

Gene expression analysis identified a 35% decrease in ERβ mRNA expression in the middle frontal gyrus of ASD subjects. In addition, a 38% reduction in aromatase (CYP19A1) mRNA expression was observed in ASD subjects. We also found significant decreases in ER co-activators that included a 34% decrease in SRC-1, a 77% decrease in CBP, and a 52% decrease in P/CAF mRNA levels in ASD subjects relative to controls. There were no differences in the mRNA levels of TIF-2, AIB-1 (ER co-activators), ER co-repressors (SMRT and nCoR) and ERα in the middle frontal gyrus of ASD subjects as compared to controls. We observed significant correlations between ERβ, CYP19A1, and co-activators in the study subjects. Immunoblot analysis further confirmed the changes in ERβ and aromatase at the protein level in the control and ASD subjects.

**Conclusions:**

These results, for the first time, provide the evidence of the dysregulation of ERβ and co-factors in the brain of subjects with ASD.

**Electronic supplementary material:**

The online version of this article (doi:10.1186/2040-2392-5-46) contains supplementary material, which is available to authorized users.

## Background

Autism spectrum disorders (ASD) are a heterogeneous set of neurodevelopmental disorders including autism, Asperger’s syndrome, childhood disintegrative disorder, and pervasive developmental disorder not otherwise specified (PDD-NOS). Autism is currently diagnosed by extensive behavioral and psychological testing and diagnosis is declared based on DSM-V characteristics. These characteristics include communication deficits, excessive dependence on routine, and obsessive tendencies showing up in early childhood. ASDs are more prevalent in boys than in girls, with ratios of 3:1 for classic autism and 10:1 for Asperger syndrome, suggesting the possible role of sex hormones in the pathophysiology of this disorder [1]. It has been suggested that high levels of testosterone during early development may be a risk factor for ASDs [2, 3]. The above hypothesis has been supported by a number of studies reporting an association between fetal testosterone levels and autistic features [2].

Estradiol, the most potent estrogen, is formed from testosterone by the enzyme aromatase, also known as cytochrome P450, family 19 (CYP19A1). Estrogen is neuroprotective and plays an important role in emotional responses [4] and in frontal cortical activity during cognitive task performance in humans [5]. Estrogen acts through the binding to its receptor called estrogen receptor (ER). ER exists in two main forms, ERα and ERβ, which have distinct tissue expression patterns [6]. ERα and ERβ are encoded by separate genes, *ESR1* and *ESR2*, respectively, found at different chromosomal locations, and numerous mRNA splice variants exist for both receptors in both diseased and normal tissue [7]. In the “classical” pathway of estrogen action, estrogen binds to ER, a ligand-activated transcription factor that regulates transcription of target genes in the nucleus by binding to estrogen response element regulatory sequences in target genes and recruiting co-regulatory proteins such as co-activators or co-repressors. The major co-regulators involved in estrogen signaling are steroid receptor co-activator 1 (SRC-1), transcriptional mediators/intermediary factor 2 (TIF-2), nuclear receptor co-repressor 1 (nCoR), CREB-binding protein (CBP), p300/CREB-binding protein-associated protein (P/CAF), amplified in breast 1 (AIB-1), and silencing mediator of retinoid and thyroid hormone receptors (SMRT) [8]. Among these co-factors, SRC-1, TIF-2, CBP, AIB-1, and P/CAF are transcriptional co-activators, whereas SMRT and nCoR are transcriptional repressors. On the other hand, in the rapid or “non-genomic” pathway, activation of the membrane ER by estrogen leads to a rapid change in various intracellular signaling molecules including kinases, which in turn regulate gene transcription [9].

ERα is widely distributed in the various brain regions including amygdala-hippocampal area, periamygdaloid cortex, and posterior cortical nucleus of the brain [10]. Moreover, ERα influences various neurotransmitter systems, such as dopamine, serotonin (5-HT), and norepinephrine, indicating its role in neuropsychiatric disorders [11, 12]. ERβ is the principal estrogen receptor expressed in brain areas such as cerebral cortex, hippocampus, and cerebellum [13]. An earlier study has found a significant association of the ERβ gene with scores on the Autism Spectrum Quotient and the Empathy Quotient in ASD subjects [14]. Moreover, ERβ mediates some of the effects of estrogens on anxiety, locomotor activity, fear responses, and learning behavior [15]. Although the above studies are interesting, it is still unclear whether the expression of ER is impaired in the brain of ASD subjects. In the present study, we first examined the gene expression of ERα, ERβ, CYP19A1, and the co-regulators SRC-1, TIF-2, CBP, P/CAF, SMRT, AIB-1, and nCoR in postmortem middle frontal gyrus of ASD and control subjects. The middle frontal gyrus region was selected because a number of studies from neurocognitive as well as neuroimaging studies have implicated middle frontal gyrus in the pathophysiology of ASD [16–18]. Moreover, a sexual dimorphic nature has been reported in the middle frontal gyrus [19]. Based on our mRNA data, we then used western blotting to examine whether the changes found at the gene level of ERβ and CYP19A1 are significant at the protein level. We hypothesized that the expression of ERs are impaired in ASD and these abnormalities involve key co-regulators involved in ER regulation.

## Methods

### Ethics statement

The Georgia Regents University Institutional Review Board has deemed this study exempt from full review due to the use of de-identified human postmortem brain samples, with no possibility to track back the identity of the donors. There was no involvement of animal studies in this paper.

### Postmortem brain tissues

Postmortem middle frontal gyrus tissues of ASD (n = 13) and control (n = 13) subjects were received from the NICHD Brain and Tissue Bank for Developmental Disorders at the University of Maryland, Baltimore, MD, USA. Table 1 shows a detailed description on the demographics of samples. The individual scores for each of the symptomatic domains, Autism Diagnostic Interview-Revised (ADI-R) were obtained from the brain bank website. The information on ADI-R was available for 9 out of 13 subjects with ASD. We did not find any significant difference in confounding variables such as PMI, refrigeration interval, age at death, RNA integrity, and brain pH between ASD and control subjects.

**Table 1:** **Demographic characteristics of postmortem brain samples**

Variable	Control	ASD
Age (years)	11.70 ± 1.584	11.80 ± 1.609
PMI (h)	14.46 ± 2.171	19.00 ± 2.776
Sex (F/M)	1/12	0/13
Storage (days)	4287 ± 638.7	2829 ± 397.7
RIN	5.55 ± 0.71	6.84 ± 0.53
pH	5.95 ± 0.06	6.11 ± 0.07
Manner of death	Drowning (3), Vehicle accident (4), Hanging/suicide (2), Cardiovascular complication (1), TSS (1), Multisystem failure (1), Anaphylaxis (1)	Drowning (6)
		Vehicle accident (1)
		Cardiovascular complication (2)
		Cancer (1)
		Hemorrhage (2)
		Diabetic ketoacidosis (1)
Medications*	Yes (1) (Concerta, Clonidine)	Yes (4) (Zyprexa (1), Reminyl (1), Naltrexone (1), Risperdal (2), Luvox (1), Clonidine (1), Insulin (1))
	No (12)	
		No (9)

ASD, Autism spectrum disorder; F, Female; M, Male; PMI, Postmortem interval; RIN, RNA integrity; TSS, Toxic shock syndrome. Values are Mean ± SE. *Note that some individuals took multiple medications.

### Immunoblotting

Brain tissue was homogenized in a tissue lysis buffer containing 50 mM Tris-HCl (pH 7.5), 150 mM NaCl, 1.0% sodium deoxycholate, 0.1% sodium dodecyl sulfate (SDS), 2 mM EDTA, 6 μM PMSF, and 1.0% Triton X-100 supplemented with protease inhibitor cocktail (Sigma). The homogenate was centrifuged at 13,000 rpm for 10 min at 4°C and the supernatant was used for protein estimation by the bicinchoninic acid method (BCA Protein Assay Kit, Sigma). Samples (30–40 μg) were subjected to SDS-PAGE and transferred onto a nitrocellulose membrane. The membrane was blocked for 1 h in PBS with Tween 20 and 5% non-fat milk or 5% BSA followed by overnight incubation with a primary antibody. The primary antibodies used were: anti-ERβ (1:2,000, Abcam; ab3577 [20, 21]; anti-ERα (1:500, Santa Cruz Biotech; sc-71064); anti-aromatase (1:500, Santa Cruz Biotech; sc-14245); anti-CBP (1:200, Santa Cruz Biotech; sc-7300); anti-SRC-1 (1:500, Santa Cruz Biotech; sc-32789), or anti-P/CAF (1:500, Santa Cruz Biotech; sc-13124). Following washing, the membranes were incubated with secondary antibody for 1 h. We used enhanced chemiluminescence detection reagent kit (Thermo Scientific) to detect the proteins. The intensity of the bands was quantified using densitometry software (Image J, NIH). The immunoblot data was corrected for corresponding glyceraldehyde 3-phosphate dehydrogenase (GAPDH; 1:5,000, Cell Signaling) values. For immunoprecipitation, 300 μg of proteins were pre-cleared for 2 h with 40 μL of PureProteome Protein A and G Magnetic Beads (Millipore) and 40 μg IgG antibody (Millipore), followed by incubation overnight with the primary antibody. The immunoprecipitated proteins were subjected to immunoblotting for the detection of co-precipitated proteins.

### Quantitative reverse transcriptase PCR (qRT-PCR)

Total RNA from postmortem brain tissues was isolated using a commercially available kit (SV RNA Isolation, Promega, Madison, WI, USA). qRT-PCR was performed on a MasterCycler (Eppendorf) using a SuperScript III Platinum SYBR Green One-Step qRT-PCR kit (Invitrogen, Carlsbad, CA, USA). A typical reaction mixture of a total volume of 25 μL consisted of 0.5 μL Superscript III RT/Platinum Taq mix, 12.5 μL 2X SYBR Green Reaction Mix (includes 0.4 mM of each dNTP and 6 mM MgSO_4_), 12.5 pMol of each of forward or reverse primers, and 4 μL DEPC-treated water. PCR amplification was done with an initial incubation at 55°C for 1,200 sec, then at 95°C for 120 sec followed by 35 cycles of 95°C for 15 sec, 50°C for 30 sec, 72°C for 30 sec, and a final melting curve from 55°C to 95°C at 0.2°C/sec. We confirmed the primer specificity by melting curve analysis and electrophoresis of PCR products on a 2% agarose gel to confirm the presence of a single band of the predicted size. The mRNA for genes of interest was normalized to two control genes (GAPDH and *β*-actin) and a geometric mean of these genes. The mRNA expression levels were quantified by the delta-delta Ct method. Primers were synthesized by Integrated DNA Technologies (Additional file 1: Table S1).

### Statistical analysis

Analysis of covariance (ANCOVA) models were used to examine the differences in estrogen receptor expression between postmortem samples of people with ASD and the control sample (i.e., affection status). To examine the unique effects of affection status on estrogen receptor, age, postmortem interval, storage time, sample pH, and RNA integrity number were added to the model as covariates. Only covariates with at least small associations with an estrogen receptor expression were considered for inclusion in the ANCOVA model with the receptor signal as a dependent variable. Candidate covariates that were significantly correlated with receptor expression levels were included in the model. To maximize the observed power, non-significant covariates were removed from the model until only significant covariates remained in the model; η^2^ and partial η^2^ coefficients were computed as estimates of effect size. Exact probability (*P*) values of less than 5% were considered significant. Simple correlations were computed to examine the association of covariates and clinical variables with receptor expression levels. All analyses were performed using SPSS Statistics 20 software (IBM).

## Results

The postmortem sample comprised 26 subjects, half of whom had a confirmed diagnosis of an ASD and half of whom were age- and gender-matched controls.

### Decrease in mRNA levels of ERβ and CYP19A1, but no change in ERα mRNA in the middle frontal gyrus of ASD subjects

**Figure 1:**
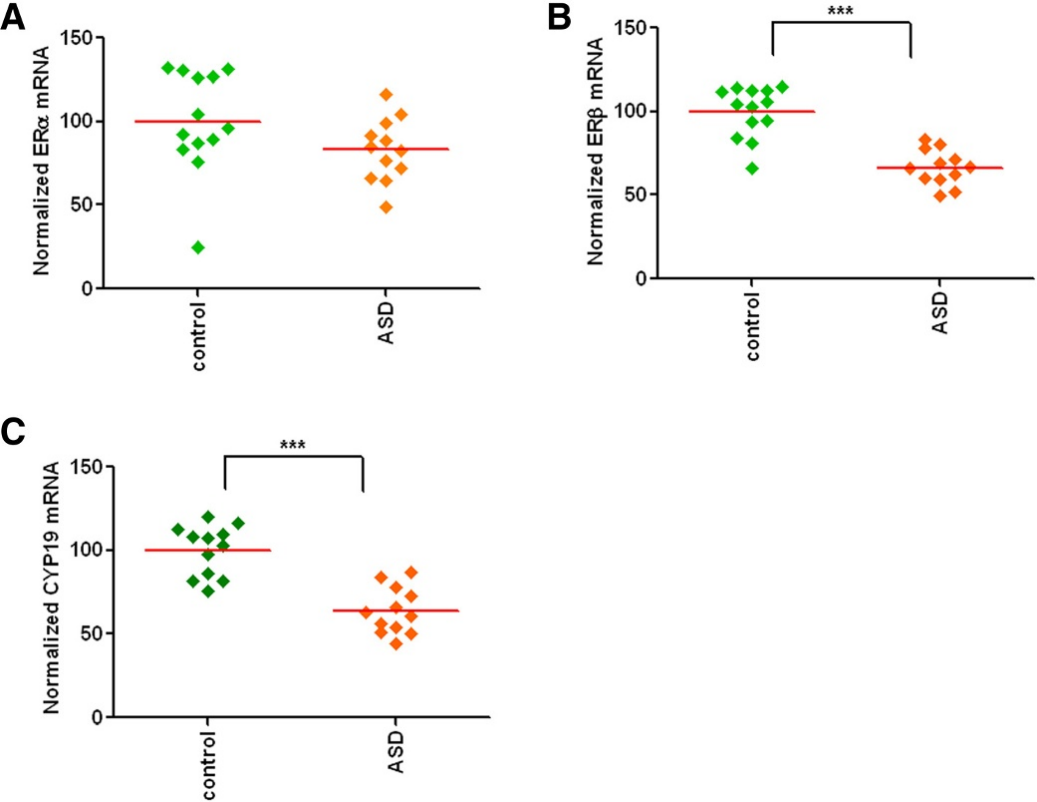
**Decrease in mRNA levels of ERβ and CYP19A1, but no change in ERα mRNA in the middle frontal gyrus of ASD subjects.** **(A)** No significant change in ERα mRNA. **(B)** Significant reduction in ERβ mRNA in the ASD subjects. **(C)** CYP19A1 mRNA levels were significantly lower in the ASD subjects. mRNA levels were determined by qRT-PCR, and the values were normalized to the geometric mean of two control genes (GAPDH and *β*-actin). ***P <0.001 vs. controls.

The mRNA levels of ERα, ERβ, and CYP19A1 in the middle frontal gyrus of control and ASD subjects were determined by qRT-PCR. Additional file 2: Table S2 depicts the correlations between the mRNA transcripts and confounding variables. An ANCOVA [between-subjects factor: affection status (ASD, Control); covariates: age, postmortem interval, storage time, sample PH] was performed in ERα data analysis. None of these covariates demonstrated significant main effects in the prediction of ERα expression. The main effect of affection status on ERα was not statistically significant (Figure 1A) [*F*(1, 19) = 2.65, *P* = 0.12, η^2^_p_ = 0.123]. We found a statistically significant main effect of affection status on ERβ mRNA levels when age, storage time, and RNA integrity number were considered in the model as covariates (Figure 1B) [*F*(1, 20) = 34.10, *P* <0.0001, η^2^_p_ = 0.630]. None of the covariates demonstrated a significant main effect on ERβ mRNA. Subjects with ASD (estimated marginal means (EMM) = 65.85, standard error (SE) = 4.06) demonstrated much lower expression (35% decrease) of ERβ than the control group (EMM = 100.59, SE = 3.88). For CYP19A1 mRNA data analysis, age, storage time, sample pH, RNA integrity number, and postmortem interval were entered into the model as covariates. None of the covariates in the model demonstrated a significant main effect in the prediction of CYP19A1 mRNA when they were entered in the model with affection status. Affection status was a significant predictor of CYP19A1 expression (Figure 1C) [*F*(1, 17) = 27.90, *P* <0.0001, η^2^_p_ = 0.621]. Subjects with ASD had reduced (EMM = 62.76, SE = 4.78) CYP19A1 mRNA levels (38% decrease) relative to the control group (EMM = 101.29, SE = 4.78).

Table 2 shows the correlation of mRNA transcripts with ADI-R scores. We have adjusted the *P* values using the Holm-Bonferroni sequential correction for multiple comparisons. We did not find any significant correlation of ERα, ERβ, and CYP19A1 mRNA levels with any of the ADI-R scores.

**Table 2:** **Correlations of mRNA transcripts with ADI-R scores**

		Social interaction	Verbal communication	Non-verbal communication	Stereotyped behavior	Abnormality of development
**ER co-repressors**						
nCoR	r	–0.021	–0.202	0.479	–0.791	0.063
	*P*	0.961	0.745	0.277	**0.019**	0.893
	Adjusted *P*	1.000	1.000	1.000	0.095	1.000
SMRT	r	0.013	–0.090	–0.258	–0.489	–0.640
	*P*	0.976	0.866	0.576	0.219	0.122
	Adjusted *P*	1.000	1.000	1.000	0.876	0.610
**ER co-activators**						
CBP	r	–0.549	0.117	0.023	–0.261	–0.718
	*P*	0.126	0.825	0.956	0.498	**0.045**
	Adjusted *P*	0.504	1.000	1.000	1.000	0.225
P/CAF	r	–0.126	0.540	–0.319	0.515	–0.459
	*P*	0.747	0.268	0.441	0.156	0.253
	Adjusted *P*	1.000	1.000	1.000	0.780	1.000
TIF-2	R	–0.103	0.687	–0.217	0.536	–0.325
	*P*	0.792	0.132	0.606	0.137	0.432
	Adjusted *P*	1.000	0.660	1.000	0.660	1.000
SRC-1	r	–0.109	0.065	–0.876	0.294	–0.774
	*P*	0.798	0.917	**0.010**	0.480	**0.041**
	Adjusted *P*	1.000	1.000	**0.049**	1.000	0.164
AIB-1	r	0.274	0.200	0.099	-0.582	-0.000
	*P*	0.475	0.703	0.814	0.099	0.999
	Adjusted *P*	1.000	1.000	1.000	0.495	1.000
**Estrogen receptor**						
ERα	r	0.081	–0.210	0.265	–0.157	0.505
	*P*	0.848	0.735	0.566	0.710	0.248
	Adjusted *P*	1.000	1.000	1.000	1.000	1.000
ERβ	r	0.112	–0.203	0.192	–0.215	0.316
	*P*	0.791	0.743	0.680	0.610	0.489
	Adjusted *P*	1.000	1.000	1.000	1.000	1.000
**Enzyme**						
CYP19A1	r	0.405	–0.746	–0.143	–0.227	0.148
	*P*	0.320	0.148	0.760	0.589	0.751
	Adjusted *P*	0.740	1.000	1.000	1.000	1.000

ADI-R scores were available for only 9 of the 13 ASD subjects. Adjusted *P* denotes *P* values adjusted using the Holm-Bonferroni sequential correction for multiple comparisons; r, Pearson correlation; *P*, level of significance. Bolded text indicates significant *P* values.

### Decrease in mRNA levels of ER co-activators (SRC-1, CBP and P/CAF), but no change in ER co-repressors (SMRT and nCoR) in the middle frontal gyrus of ASD subjects

**Figure 2:**
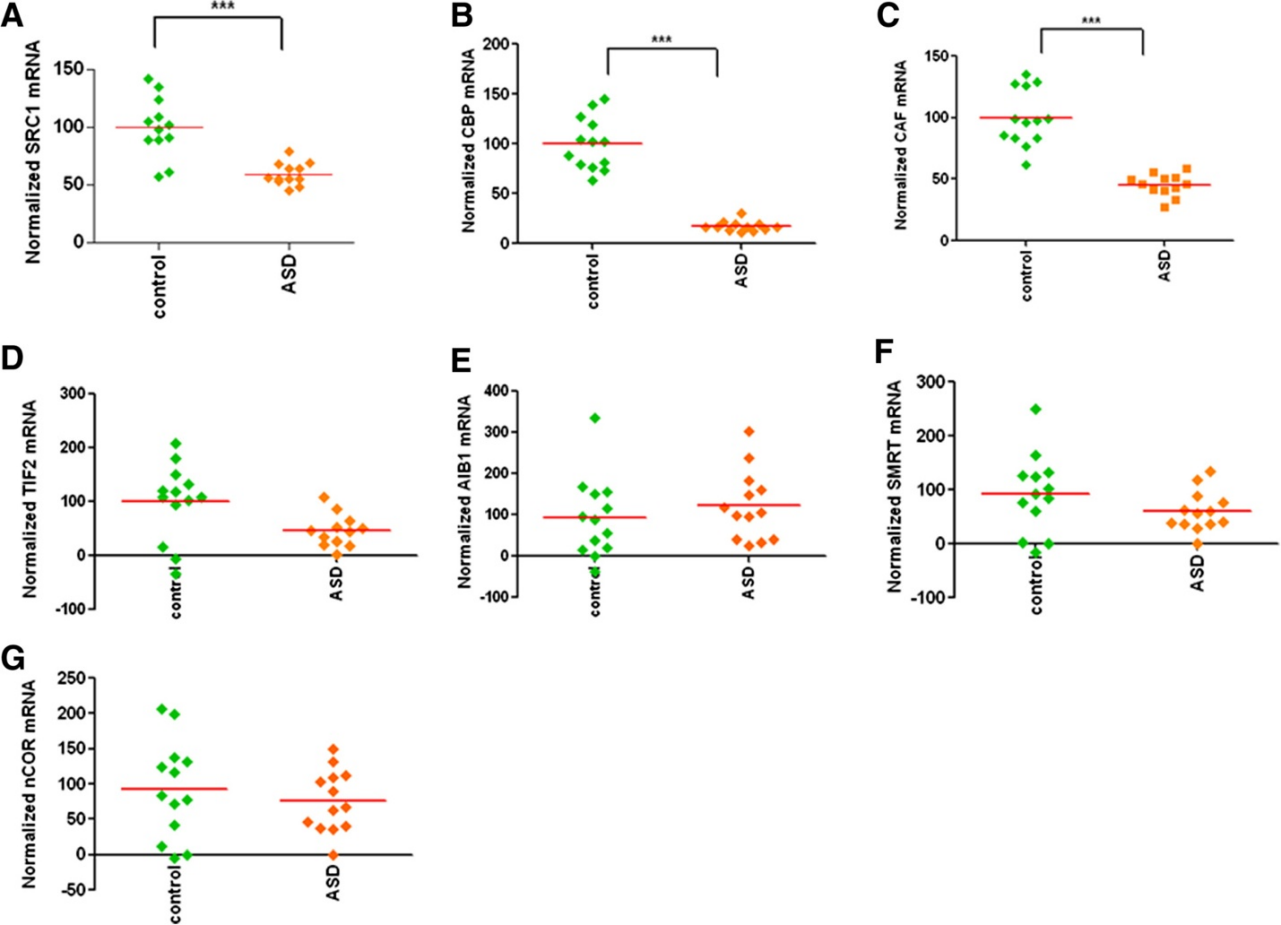
**Decrease in mRNA levels of ER co-activators (SRC-1, CBP, and P/CAF), but no change in ER co-repressors (SMRT and nCoR) in the middle frontal gyrus of ASD subjects.** **(** **A–E** **)** Normalized mRNA expression of ER co-activators: **(** **A** **)** SRC-1, **(** **B** **)** CBP, **(** **C** **)** P/CAF, **(** **D** **)** TIF-2, and **(** **E** **)** AIB-1. **(** **F** **and** **G** **)** Normalized mRNA expression of ER co-repressors: **(** **F** **)** SMRT and **(** **G** **)** nCoR. mRNA levels were determined by qRT-PCR, and the values were normalized to the geometric mean of two control genes (GAPDH and *β*-actin). ***P <0.001 vs. controls.

Postmortem interval, storage time, sample pH, and RNA integrity number were entered as covariates in the model to analyze SRC-1 mRNA data. None of the covariates in the model demonstrated a significant main effect when they were entered in the model with affection status. The predicted main effect of affection status on SRC-1 expression was statistically significant (Figure 2A) [*F*(1, 18) = 11.05, *P* = 0.004, η^2^_p_ = 0.380]. We found a significant reduction in SRC-1 mRNA (34% decrease) in the middle frontal gyrus of subjects with ASD (EMM = 63.33, SE = 6.26) as compared to controls (EMM = 95.85, SE = 6.26). The predicted main effect of affection status on CBP mRNA was statistically significant, when postmortem interval, storage time, sample pH, and RNA integrity number were entered as covariates in the model (Figure 2B) [*F*(1, 18) = 53.15, *P* <0.0001, η^2^_p_ = 0.747]. None of the covariates in the model achieved statistical significance. Overall, subjects with ASD (EMM = 21.86, SE = 6.65) demonstrated much lower expression (77% decrease) of CBP than the control group (EMM = 95.64, SE = 6.33). Data on P/CAF mRNA was analyzed by considering postmortem interval, storage time, sample pH, and RNA integrity number as covariates in the model. However, none of these covariates remained significant predictors when they were entered into the model with affection status as an independent variable. The main effect of affection status on P/CAF expression was found significant (Figure 2C) [*F*(1, 18) = 33.53, *P* <0.0001, η^2^_p_ = 0.651]. Moreover, a significant reduction in P/CAF mRNA expression (52% decrease) was found in the middle frontal gyrus of subjects with ASD (EMM = 47.47, SE = 5.71) as compared to controls (EMM = 97.80, SE = 5.44). Storage time, sample pH, and RNA integrity number were included in the model as covariates to analyze the mRNA data on TIF-2. Among these covariates, storage time [*F*(1, 20) = 4.70, *P* = 0.042, η^2^_p_ = 0.19] and sample pH [*F*(1, 20) = 4.32, *P* = 0.05, η^2^_p_ = 0.18] remained significant covariates in the model. The predicted main effect of affection status on TIF-2 expression did not achieve statistical significance (Figure 2D) [*F*(1, 20) = 1.49, *P* = 0.236, η^2^_p_ = 0.07]. Similarly, no significant effect of affection status was observed on AIB-1 mRNA expression (Figure 2E) ([*F*(1, 20) = 1.37, *P* = 0.264, η^2^_p_ = 0.06].

Next, we examined whether the mRNA levels of ER co-repressors (SMRT and nCoR) are altered in the postmortem brain tissues of subjects with ASD. With postmortem interval and storage time entered as covariates in the model, the predicted main effect of affection status on SMRT expression was not statistically significant (Figure 2F) [*F*(1, 17) = 0.013, *P* = 0.91, η^2^_p_ = 0.001]. We found that storage time [*F*(1, 17) = 7.51, *P* = 0.014, η^2^_p_ = 0.306] remained a statistically significant covariate in this model. Data on nCoR mRNA was analyzed by considering postmortem interval, storage time, sample pH, and RNA integrity number as covariates in the model. No main effect of affection status was found on nCoR expression (Figure 2G) [*F*(1, 17) = 0.046, *P* = 0.83, η^2^_p_ = 0.003]. Storage time [*F*(1, 17) = 9.59, *P* = 0.007, η^2^_p_ = 0.361] was the only statistically significant covariate found in this model.

We observed some significant associations between the co-factors and the ADI-R scores in the subjects with ASD (Table 2). SRC-1 mRNA was negatively correlated with non-verbal communication (r = -0.876, *P* = 0.01) and abnormality of development (r = -0.774, *P* = 0.041) scores. A significant negative correlation was found between CBP mRNA and abnormality of development score (r = -0.718, *P* = 0.045). In addition, nCoR mRNA was negatively associated with stereotyped behavior score (r = -0.791, *P* = 0.019).

### Correlations between mRNA expression of ERs, CYP19A1, and co-factors in the middle frontal gyrus of study subjects

We used Spearman’s correlation to examine the relationships between mRNA expression of ERs, CYP19A1, and co-factors in the middle frontal gyrus of the study subjects. Additional file 3: Table S3 depicts the correlations between the mRNA transcripts. ERα was positively correlated with ERβ and CYP19A1 mRNA levels. A large positive correlation was observed between ERβ and CYP19A1. In addition, ERβ was positively correlated with SRC--1, P/CAF, and CBP. Similarly, CYP19A1 was positively correlated with SRC1, P/CAF, and CBP. We also found significant correlations in the mRNA levels between the co-factors. Positive associations were found between SRC1 and P/CAF, CBP, TIF-2, SMRT, or nCoR. TIF-2 was positively correlated with P/CAF and CBP. In addition, we observed positive correlations between P/CAF and CBP mRNA levels, and CBP with SMRT expression. Interestingly, a significant positive correlation was also found in the mRNA levels between the co-repressors SMRT and nCOR. We did not find any significant correlation between AIB-1 and ERs, CYP19A1, or other co-factors examined in this study.

### Decrease in ERβ and CYP19A1 protein levels in the middle frontal gyrus of ASD subjects

**Figure 3:**
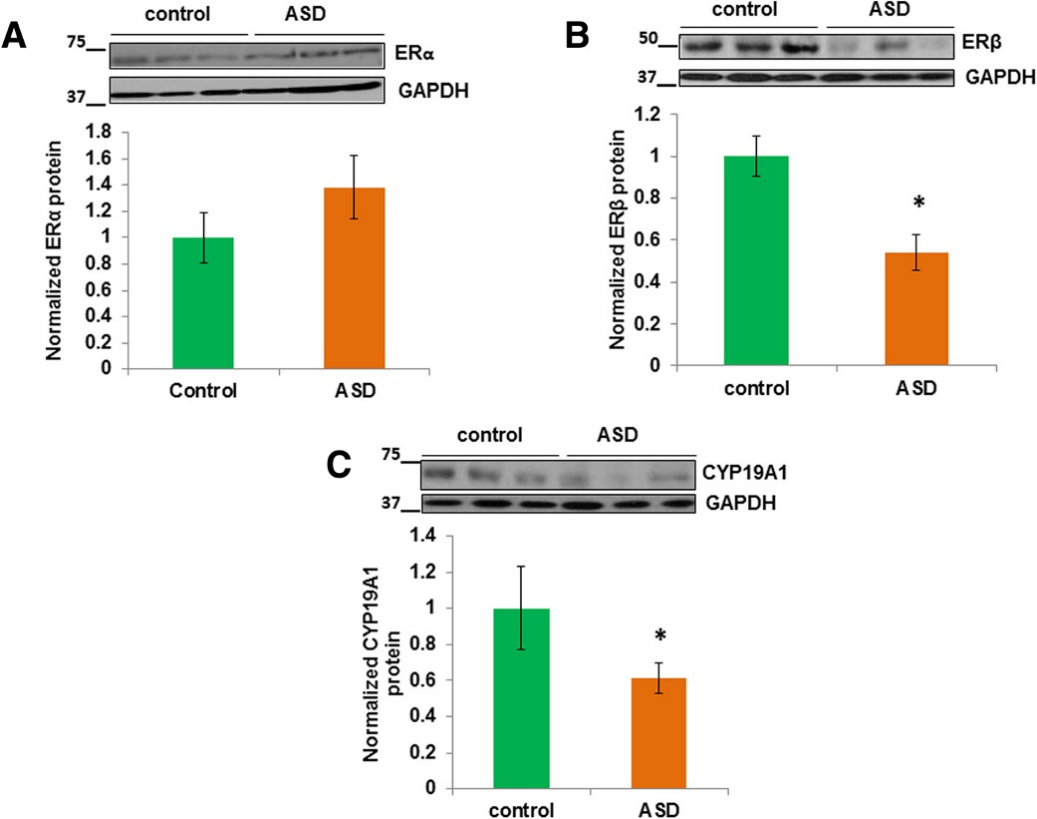
**Decrease in ERβ and CYP19A1 protein levels in the middle frontal gyrus of ASD subjects.** ERα, ERβ, and CYP19A1 protein levels were determined by western blot analysis. The upper panels show representative autoradiogram of **(** **A** **)** ERα, **(** **B** **)** ERβ, or **(** **C** **)** CYP19A1 and GAPDH, and the lower panels represent fold-change in normalized ERα, ERβ, and CYP19A1 protein levels, respectively. Results are mean ± SEM vs. controls. *P <0.05 vs. controls.

Given that we found significant reductions in ERβ and CYP19A1 mRNA levels in the middle frontal gyrus of subjects with ASD, we next determined the expression of the ERα, ERβ, and CYP19A1 at the protein level. We did not find any significant change in ERα protein levels in the middle frontal gyrus of subjects with ASD as compared to controls (Figure 3A). We found a significant reduction in ERβ protein (Figure 3B; *P* <0.05) and CYP19A1 (Figure 3C; *P* <0.05) levels in the middle frontal gyrus of subjects with ASD as compared to controls. Next, we examined whether the co-factors whose levels were altered in the ASD interact with ERβ in the brain samples. We performed immunoprecipitation assays to determine possible conjugation of P/CAF, SRC-1, and CBP with ERβ in the middle frontal gyrus of control subjects, and each of these immunoprecipitates was examined for co-purification of ERβ by western blot. ERβ was detected in P/CAF, SRC-1, or CBP immunoprecipitates, but not in the control IgG (Additional file 4: Figure S1).

## Discussion

We have found, for the first time, alterations in ERβ transcriptional regulation in the brain of ASD subjects. We also report a significant decrease in CYP19A1 expression in ASD subjects. The above changes were associated with alterations in ER co-activators in the same study subjects. We did not find a significant difference in ERα expression between ASD and controls. Together, these findings suggest alterations in ER signaling in ASD.

The decrease in ERβ mRNA and protein expression in the middle frontal gyrus of ASD subjects found in this study is consistent with the increasing evidence for the role of estrogen signaling in the etiology of ASD. A significant association of the ERβ gene with autism traits as measured by the Autism Spectrum Quotient and the Empathy Quotient has been reported in ASD subjects [14]. ERβ is abundantly expressed in the cortex [22]. Moreover, ERβ plays an important role in neurodevelopment, and ERβ knockout, but not ERα knockout mice show defects of neuronal migration [23]. It is known that ERα and ERβ have distinct tissue expression profiles, and have different cellular functions [22–24]. ERα is involved in mediating estrogen action on reproductive organs and reproductive behavior, whereas ERβ is known to mediate some of the effects of estrogens on behaviors that are not specifically associated with reproduction, such as locomotor activity, fear responses, anxiety, and learning [15]. ERβ knockdown has been shown to abolish E2-induced reductions in depressive behavior in mice [25–27]. Moreover, administration of ERβ agonist or selective ligand has been shown to reduce anxiety-type behavior [27] and depressive behavior [28] in rats. Activation of ERβ with the specific agonist WAY-200070 in cortical neurons results in increased spine density and PSD-95 (postsynaptic density-95) accumulation in membrane [29]. Together, these results suggest that ERβ-mediated mechanism(s) are important for E2-induced neuronal plasticity.

The decrease in ERβ expression found in the ASD subjects might be the result of transcriptional regulation, either through methylation or by the regulation of genes of specific transcription factors binding to the ERβ promoter. Hypermethylation of the ERβ promoter is associated with a marked decrease in ERβ mRNA expression [30]. It is known that estrogen binds to ER leading to a conformational change in ER. The estrogen-ER complex can bind directly to DNA via an estrogen responsive element or become attached to a transcription factor [24]. It recruits a variety of co-regulators that result in the activation or repression of target genes by modifying chromatin structure. The p160/SRC (steroid receptor co-activator) family is one of the most studied classes of co-activators [31]. Among the co-activators, SRC-1 and CBP exhibit autonomous histone acetyltransferase activity that promotes efficient transcription. In contrast, in the absence of ligands, ER associates with co-repressors nCoR or SMRT to mediate transcriptional repression of target genes through the histone deacetylase activity of the co-repressors [32–34]. The present study revealed a novel finding that ERβ expression levels correlated with ER co-activators, SRC-1, P/CAF, and CBP. Moreover, we observed that the above co-factors interact with ERβ in human middle frontal gyrus. It is known that ERβ can antagonize ERα-dependent transcription in cells [35]. In addition, ERβ and its variant, ERβ2, have been shown to increase the proteolytic degradation of ERα [36]. Thus, it is possible that the changes in the expression levels of ERs and co-factors observed in our study might influence the estrogen receptor signaling machinery and might play important roles in the pathophysiology of ASD.

Our findings demonstrate that CYP19A1 expression is significantly lower in the brain of ASD subjects. Earlier studies have reported the expression of CYP19A1, the key enzyme required for estrogen production, in the cortex [37]. Furthermore, CYP19A1 is enriched at synapses and localizes to presynaptic structures in cortical neurons [29], suggesting that brain-synthesized estrogen plays an important role in neuronal function [38]. The decrease in CYP19A1 could lead to reduced conversion of testosterone to estradiol resulting in increased levels of testosterone as observed in ASD subjects [39]. Our data is in agreement with a previous finding on reduced aromatase protein levels in the frontal cortex of ASD subjects [40]. An earlier genetic study has reported association between androgen receptor and ASD, suggesting an important role of androgen signaling in ASD [41]. Further studies should examine the mRNA and protein levels of androgen receptors in the brain samples from ASD and control subjects, and such information would be helpful to better understand the relationship between estrogen-related and testosterone-related signaling pathways in ASD.

## Conclusions

We have identified dysregulation of ERβ, CYP19A1, and co-activators associated with ER signaling in the middle frontal gyrus of ASD subjects with a significant association between these molecules. Our data suggest that a coordinated regulation of ER signaling molecules plays an important role in ER signaling in the brain, and that this network may be impaired in subjects with ASD. Although we found a large, significant association between the co-factor mRNA transcripts (SRC-1, CBP, and nCoR) and ADI-R scores in ASD subjects, its implication is unclear. Moreover, the robust reduction found in CBP mRNA expression in ASD subjects needs further investigation. CBP is known to be associated with other steroid receptors, including progesterone receptor, thyroid hormone receptors, and retinoid receptors [32]. It is important to examine whether the complex formation of ERβ with CBP is indispensible for ER-dependent neuronal plasticity and ASD-like behavior. Moreover, the present data was collected in a relatively smaller number of study subjects, which needs further investigation using large samples before a conclusion can be drawn. Since brain tissue from individuals with ASD is quite scarce, lymphoblastoid cell lines that are banked for ASD cohorts (though there are several limitations including difference in tissue type and difficulties in transformation procedure) could provide a large sample of biological material to understand the pathophysiology of ASD. Future studies will investigate the mechanism of regulation of ERβ in ASD, which might lead to a better understanding of the pathophysiology and provide new avenues of treatment of this disorder.

### Availability of supporting data

The data sets supporting the results of this article are included within the article and its additional files.

## Electronic supplementary material

Additional file 1: Table S1: List of primers used in the qRT-PCR. (DOCX 15 KB)

Additional file 2: Table S2: Correlations of mRNA transcripts with confounding variables. (DOCX 15 KB)

Additional file 3: Table S3: Correlations between mRNA transcripts. (DOCX 21 KB)

Additional file 4: Figure S1: ERβ is associated with the co-factors, P/CAF, SRC1, or CBP. Lysates from postmortem middle frontal gyrus of control subjects were subjected to immunoprecipitation (IP) using a co-factor antibody followed by western blotting (WB) with the ERβ or co-factor antibody. A separate IP assay was performed for (A) P/CAF, (B) SRC1, or (C) CBP. Lysate represents 10% of the amount used in the IP. IgG, IgG control. (TIFF 56 KB)

## Abbreviations

ADI-R: Autism diagnostic interview revised
AIB-1: Amplified in breast 1
ASD: Autism spectrum disorders
CBP: CREB-binding protein
CYP19A1: Cytochrome P450, Family 19, Subfamily A, Polypeptide 1
EMM: Estimated marginal means
ER: Estrogen receptor
ERα: Estrogen receptor alpha
ERβ: Estrogen receptor beta
GAPDH: Glyceraldehyde 3-phosphate dehydrogenase
nCoR: Nuclear receptor co-repressor 1
P/CAF: p300/CREB-binding protein-associated protein
SE: Standard error
SMRT: Silencing mediator of retinoid and thyroid hormone receptors
SRC-1: Steroid receptor co-activator 1
TIF-2: Transcriptional mediators/intermediary factor 2.
