# Supplementary material for: Dysregulation of estrogen receptor beta (ERβ), aromatase (CYP19A1), and ER co-activators in the middle frontal gyrus of autism spectrum disorder subjects
Source: Mol Autism. 2014 Sep 9;5:46. doi: 10.1186/2040-2392-5-46 (PMC4161836; doi:10.1186/2040-2392-5-46)
Supplement: Supplementary file 1 — Additional file 1: Table S1: List of primers used in the qRT-PCR. (DOCX 15 KB) [file 13229_2014_137_MOESM1_ESM.docx]

**Table S1.** List of primers used in the qRT-PCR.

| **Gene** | | **Primer sequence (5’ to 3’)** |
| --- | --- | --- |
| CYP19A1-RP | | GAT TTT AAC CAC GAT AGC ACT TTC G |
| CYP19A1-FP | | CCC TTC TGC GTC GTG TCA T |
| SMRT-RP | | CGG AAT CTT CCC CTC CTC CC |
| SMRT-FP | | TGT GGT TCA TAA GCC ATC TGC |
| N-CoR-RP | | TTG GAC TCT TGG ATG TGC C |
| N-CoR-FP | | GCT GAT GAG GAT GTG GAT GG |
| P/CAF-RP | | TGC CTC AAG TCC AGA AGA GG |
| P/CAF-FP | | AGA ACA TTG CTT CGC TCG G |
| CBP-RP |  | TGT TGA ACA TGA GCC AGA CG |
| CBP-FP |  | TCA GTC AAC ATC TCC TTC GC |
| TIF2-RP |  | TCT GTG TAT GTG CCA TTC GG |
| TIF2-FP |  | TAA TGC ACA GAT GCT GGC C |
| SRC-1-RP | | ACT ACT TGT CAT GCC AAC GG |
| SRC-1-FP | | GCT CGT TCA TCC ACA TTG CC |
| AIB1-FP | | TCA TAG GTT CCA TTC TGC CG |
| AIB1-RP | | CGT CCT CCA TAT AAC CGA GC |
| ER-β-RP | | CGT AAC ACT TCC GAA GTC GG |
| ER- β -FP | | TCA CAT CTG TAT GCG GAA CC |
| ER-α-RP |  | GCC AGG CAC ATT CTA GAA GG |
| ER- α -FP |  | AGA CAT GAG AGC TGC CAA CC |
| GAPDH-FP |  | GAG TCA ACG GAT TTG GTC GT |
| GAPDH-RP |  | TTG ATT TTG GAG GGA TCT CG |
| Actin-FP |  | GGA CTT CGA GCA AGA GAT GG |
| Actin-RP |  | AGC ACT GTG TTG GCG TAC AG |
