# Supplementary material for: Dysregulation of estrogen receptor beta (ERβ), aromatase (CYP19A1), and ER co-activators in the middle frontal gyrus of autism spectrum disorder subjects
Source: Mol Autism. 2014 Sep 9;5:46. doi: 10.1186/2040-2392-5-46 (PMC4161836; doi:10.1186/2040-2392-5-46)
Supplement: Supplementary file 2 — Additional file 2: Table S2: Correlations of mRNA transcripts with confounding variables. (DOCX 15 KB) [file 13229_2014_137_MOESM2_ESM.docx]

|  | **Age** | | **PMI** | **Storage Time** | | | **pH** | **RIN** |
| --- | --- | --- | --- | --- | --- | --- | --- | --- |
| nCoR | 0.174 | -0.053 | | | 0.520* | -0.071 | | 0.165 |
| SMRT | 0.107 | -0.325 | | | 0.548* | -0.161 | | -0.022 |
| CBP | 0.046 | -0.278 | | | 0.406* | -0.340 | | -0.390 |
| P/CAF | -0.026 | -0.313 | | | 0.440* | -0.403* | | -0.153 |
| TIF2 | -0.251 | 0.042 | | | 0.439* | -0.311 | | -0.119 |
| SRC1 | -0.028 | -0.278 | | | 0.462* | -0.379 | | -0.169 |
| AIB1 | -0.042 | -0.231 | | | -0.169 | -0.262 | | -0.115 |
| Erα | -0.183 | 0.182 | | | 0.260 | 0.146 | | 0.074 |
| Erβ | -0.254 | -0.041 | | | 0.298 | -0.090 | | -0.211 |
| CYP19A1 | -0.216 | -0.127 | | | 0.196 | -0.175 | | -0.297 |

**Table S2**. Correlations of mRNA transcripts with confounding variables.

Values are Pearson Correlation. PMI = Postmortem Interval; RIN = RNA integrity; *p < 0.05
