# Supplementary material for: Dysregulation of estrogen receptor beta (ERβ), aromatase (CYP19A1), and ER co-activators in the middle frontal gyrus of autism spectrum disorder subjects
Source: Mol Autism. 2014 Sep 9;5:46. doi: 10.1186/2040-2392-5-46 (PMC4161836; doi:10.1186/2040-2392-5-46)
Supplement: Supplementary file 3 — Additional file 3: Table S3: Correlations between mRNA transcripts. (DOCX 21 KB) [file 13229_2014_137_MOESM3_ESM.docx]

|  | **nCOR** | **SMRT** | **CBP** | **P/CAF** | **TIF2** | **SRC1** | **AIB1** | **ERα** | **ERβ** | **CYP19A1** |
| --- | --- | --- | --- | --- | --- | --- | --- | --- | --- | --- |
| **nCOR** | 1 | 0.738^*^  **0** | 0.199  0.364 | 0.192  0.38 | 0.34  0.112 | 0.496^*^  **0.019** | 0.047  0.825 | 0.084  0.703 | 0.042  0.849 | 0.055  0.809 |
| **SMRT** | 0.738^*^  **0** | 1 | 0.467^*^  **0.025** | 0.301  0.163 | 0.279  0.197 | 0.557^*^  **0.007** | -0.035  0.869 | 0.306  0.155 | 0.221  0.31 | 0.141  0.533 |
| **CBP** | 0.199  0.364 | 0.467^*^  **0.025** | 1 | 0.818^*^  **0** | 0.483^*^  **0.014** | 0.787^*^  **0** | -0.310  0.131 | 0.26  0.22 | 0.665^*^  **0** | 0.589^*^  **0.003** |
| **P/CAF** | 0.192  0.38 | 0.301  0.163 | 0.818^*^  **0** | 1 | 0.606^*^  **0.001** | .800^*^  **0** | -0.211  0.309 | 0.221  0.3 | 0.568^*^  **0.004** | 0.631^*^  **0.001** |
| **TIF2** | 0.34  0.112 | 0.279  0.197 | .483^*^  **0.014** | .606^*^  **0.001** | 1 | 0.651^*^  **0.001** | -0.104  0.618 | 0.268  0.205 | 0.376  **0.07** | 0.319  0.138 |
| **SRC1** | 0.496^*^  **0.019** | 0.557^*^  **0.007** | 0.787^*^  **0** | 0.800^*^  **0** | 0.651^*^  **0.001** | 1 | -0.075  0.725 | 0.109  0.614 | 0.456^*^  **0.025** | 0.443^*^  **0.034** |
| **AIB1** | 0.047  0.825 | -0.035  0.869 | -0.310  0.131 | -0.211  0.309 | -0.104  0.618 | -0.075  0.725 | 1 | -0.076  0.719 | -0.042  0.843 | 0.167  0.435 |
| **ERα** | 0.084  0.703 | 0.306  0.155 | 0.26  0.22 | 0.221  0.3 | 0.268  0.205 | 0.109  0.614 | -0.076  0.719 | 1 | 0.752^*^  **0** | 0.503^*^  **0.012** |
| **ERβ** | 0.042  0.849 | 0.221  0.31 | 0.665^*^  **0** | 0.568^*^  **0.004** | 0.376  0.07 | 0.456^*^  **0.025** | -0.042  0.843 | 0.752^*^  **0** | 1 | 0.885^*^  **0** |
| **CYP19A1** | 0.055  0.809 | 0.141  0.533 | 0.589^*^  **0.003** | 0.631^*^  **0.001** | 0.319  0.138 | 0.443^*^  **0.034** | 0.167  0.435 | 0.503^*^  **0.012** | 0.885^*^  **0** | 1 |

**Table S3**. Correlations between mRNA transcripts. Values are Pearson Correlation; *p < 0.05.
