# Supplementary figures and images for: Dysregulation of estrogen receptor beta (ERβ), aromatase (CYP19A1), and ER co-activators in the middle frontal gyrus of autism spectrum disorder subjects
Source: Mol Autism. 2014 Sep 9;5:46. doi: 10.1186/2040-2392-5-46 (PMC4161836; doi:10.1186/2040-2392-5-46)

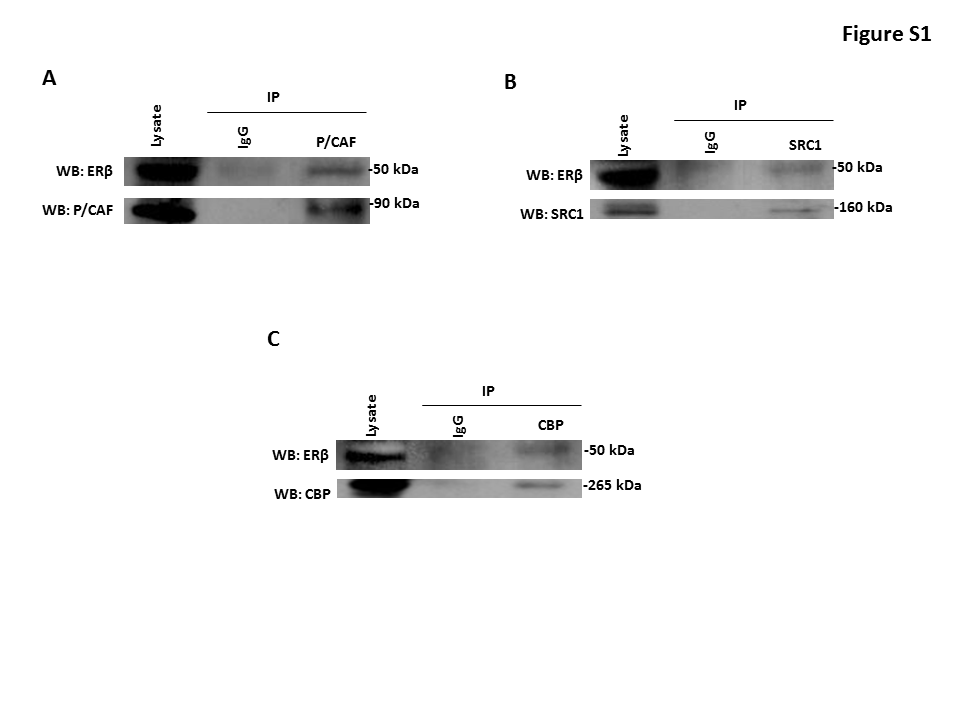

Supplement: Supplementary file 4 — Additional file 4: Figure S1: ERβ is associated with the co-factors, P/CAF, SRC1, or CBP. Lysates from postmortem middle frontal gyrus of control subjects were subjected to immunoprecipitation (IP) using a co-factor antibody followed by western blotting (WB) with the ERβ or co-factor antibody. A separate IP assay was performed for (A) P/CAF, (B) SRC1, or (C) CBP. Lysate represents 10% of the amount used in the IP. IgG, IgG control. (TIFF 56 KB) [file 13229_2014_137_MOESM4_ESM.tiff]
